# Maternal antibody uptake, duration and influence on survival and growth rate in a cohort of indigenous calves in a smallholder farming system in western Kenya^☆^

**DOI:** 10.1016/j.vetimm.2013.06.003

**Published:** 2013-09-01

**Authors:** Philip Toye, Ian Handel, Julia Gray, Henry Kiara, Samuel Thumbi, Amy Jennings, Ilana Conradie van Wyk, Mary Ndila, Olivier Hanotte, Koos Coetzer, Mark Woolhouse, Mark Bronsvoort

**Affiliations:** a The International Livestock Research Institute (ILRI), P.O. Box 30709, Nairobi 00100, Kenya; b The Roslin Institute, University of Edinburgh, Easter Bush EH25 9RG, UK; c College of Veterinary Medicine, Cornell University, Ithaca, NY 14853, USA; d Centre for Immunology, Infection & Evolution, University of Edinburgh, EH9 3JT, UK; e Department of Veterinary Tropical Diseases, Faculty of Veterinary Science, University of Pretoria, Private Bag X04, Onderstepoort 0110, South Africa; f School of Biology, University of Nottingham, Nottingham NG7 2RD, UK

**Keywords:** Colostrum, Maternal antibodies, Calves, Smallholder systems, Haemoparasites

## Abstract

The passive transfer of antibodies from dams to offspring via colostrum is believed to play an important role in protecting neonatal mammals from infectious disease. The study presented here investigates the uptake of colostrum by 548 calves in western Kenya maintained under smallholder farming, an important agricultural system in eastern Africa. Serum samples collected from the calves and dams at recruitment (within the first week of life) were analysed for the presence of antibodies to four tick-borne haemoparasites: *Anaplasma marginale*, *Babesia bigemina*, *Theileria mutans* and *Theileria parva.* The analysis showed that at least 89.33% of dams were seropositive for at least one of the parasites, and that 93.08% of calves for which unequivocal results were available showed evidence of having received colostrum. The maternal antibody was detected up until 21 weeks of age in the calves. Surprisingly, there was no discernible difference in mortality or growth rate between calves that had taken colostrum and those that had not. The results are also important for interpretation of serosurveys of young calves following natural infection or vaccination.

## 1 Introduction

The importance of the transfer of maternal antibodies into mammalian neonates via colostrum has been widely documented. This is especially important in ruminants where very little transfer of such antibody occurs *in utero* (Goddeeris, 1998). Maternal antibodies are believed to play a major role in protecting young animals from infectious disease until they acquire endogenous antibody through exposure to pathogens. Conversely, maternal antibodies can interfere with the response to infection or vaccination in young animals (Pastoret, 2007).

The IDEAL (Infectious Diseases of East African Livestock) project is a longitudinal study of 548 indigenous calves in western Kenya aimed at establishing the total infectious disease burden of these animals. The project site and study design are described in detail elsewhere (Bronsvoort et al., submitted for publication). In this smallholder system, farmers keep several species of livestock and grow different food crops. The predominant cattle breed is the Small East African Zebu. Cattle are herded in communal grazing areas or tethered at homesteads, with most farmers housing the calves separately to the adult cattle. Calves are not allowed to graze with the adults until after weaning, to prevent suckling while the dams are grazing.

The project calves were recruited within the first week of life and visited every five weeks for the following 51 weeks or until death or removal from the study. At each visit, the calves were clinically examined, and samples, including serum, were collected for later diagnostic analysis. Serum samples were also collected from the dams at the recruitment visit. These samples provide a means of studying colostral uptake in an important farming system in eastern Africa. The key questions that we wished to address were the frequency of colostral uptake on farms in the study area and the duration of maternal antibodies in individual calves. The availability of clinical, productivity and survival records of the calves permitted an assessment of the importance of colostrum uptake in the calves. In addition, the dam sera allowed us to determine the prevalence and degree of co-infections of the four parasites.

The results are important in assessing the benefits of ensuring colostral uptake in calves in smallholder farming systems where diseases represent a major constraint to productivity and the introduction of improved cattle breeds. In addition, the results demonstrating persistence of maternal antibodies are useful in interpreting seroprevalence data in young animals.

## 2 Materials and methods

### 2.1 Sampling

The samples analyzed in this study were collected as part of the IDEAL project, which monitored the presence of infectious disease in 548 indigenous calves, from birth to 12 months of age or death if before 12 months, in the Busia region of western Kenya (Bronsvoort et al., submitted for publication). This region encompasses four agroecological zones (AEZ) and stretches from Lake Victoria to Mount Elgon along the Kenya-Uganda border. The calves were selected from 20 sublocations chosen by AEZ-stratified random sampling. Recruitment occurred between October 2007 and September 2009. The calves were routinely examined for clinical signs every five weeks, and samples were taken for laboratory analysis. The calves were maintained under normal smallholder farming conditions, except that there were no therapeutic or prophylactic interventions, including acaricide application, apart from interventions on welfare grounds. Such calves were censored from the study.

The serum samples examined here were those collected from the dams and calves at the time of calf recruitment and subsequent calf samples collected every five weeks until the week 21 visit. Recruitment occurred within the first seven days after birth. Blood was drawn from the jugular vein into a plain ‘Vacutainer™’ (Becton Dickinson) tube, the serum was recovered and stored at −20 °C.

### 2.2 Serology

The sera were assayed in standard indirect ELISA for antibodies against recombinant antigens from four tick-borne haemoparasites: *Anaplasma marginale*, *Babesia bigemina*, *Theileria mutans* and *Theileria parva*. All antigens were expressed as glutathione-S-transferase fusion proteins. For *A. marginale*, the antigen was derived from a 19 kDa protein (Morzaria et al., 1999). The *B. bigemina* antigen consisted of 7 kDa of the central repeat region of the intracytoplasmic merozoite protein, p200 (Tebele et al., 2000). The *T. mutans* antigen was derived from a 32 kDa intraerythrocytic antigen (Katende et al., 1990), while the full length PIM antigen from *T. parva* Muguga (Toye et al., 1996) was used to capture antibodies to *T. parva* (Katende et al., 1998).

The assays were run in accordance with the routine protocol employed by the International Livestock Research Institute (ILRI) serology unit. In brief, the sera were added to duplicate wells coated with respective antigens. The sera were added at single dilutions of 1:40 (*A. marginale*), 1:100 (*B. bigemina* and *T. mutans*) or 1:200 (*T. parva*). Bound antibodies were detected with a mouse anti-(bovine IgG_1_) monoclonal antibody conjugated to horseradish peroxidase (Svanova, Sweden) followed by H_2_O_2_ and ABTS (2,2′-azino-di-[3-ethyl-benzothiazoline-6-sulphonic acid]) as a di-ammonium salt. The mean OD_405_ of the two wells was calculated and the percentage positivity (PP) relative to a strong positive sample was determined using the formula: PP = (OD_sample_/OD_strong positive_) × 100. Sera were considered to contain antibodies specific to the respective antigens if the PP was ≥15 (*A. marginale* and *B. bigemina*) or ≥20 (*T. mutans* and *T. parva*).

### 2.3 Data analysis

The association between calf serum antibodies (*A. marginale*, *B. babesia*, *T. mutans* and *T. parva*) at calf recruitment time and the risk for mortality and poor growth rates was tested as univariable analysis. Survival analysis and linear mixed models statistical methods were used to test the association with risk for mortality and growth rates respectively.

**Fig. 1:**
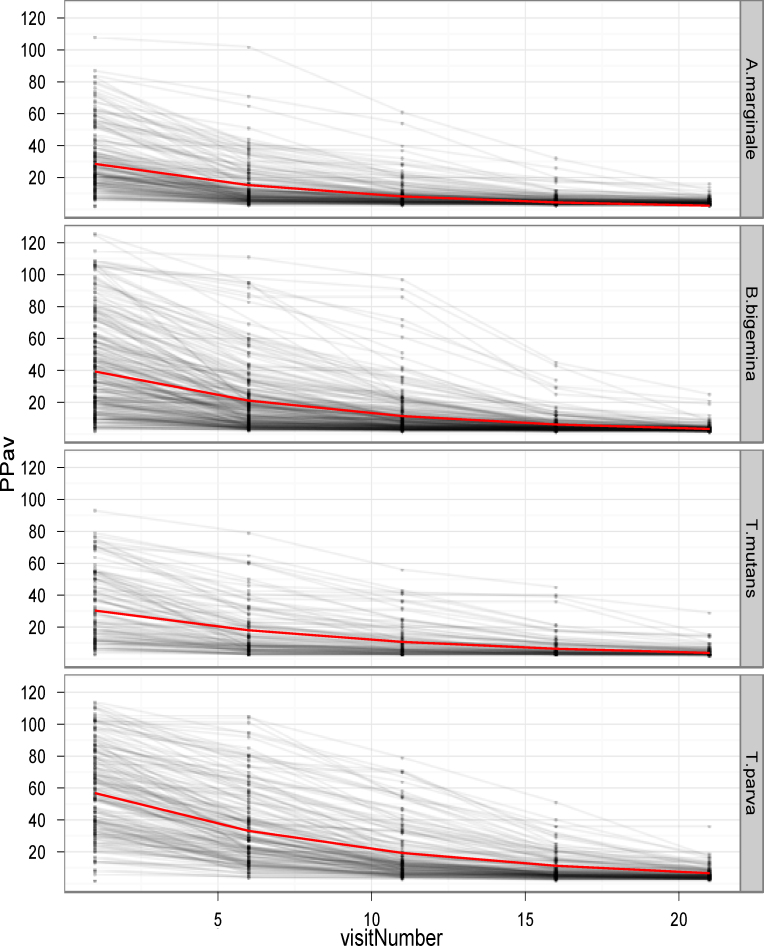
PP values over time for calves that had declining PP values up to 21 weeks. The decay curves for individual calves are shown for each infection, together with a fitted exponential decay line (red line). (For interpretation of the references to color in this figure legend, the reader is referred to the web version of the article.)

A fitted exponential decay model with random effects for calf initial starting PP with a common half-life for all calves for each parasite was used for Fig. 1.

## 3 Results and discussion

### 3.1 Antibody prevalence in dams at calving

The use of serum antibodies specific to infectious agents as an indicator of colostral uptake in calves requires a sufficient prevalence of infection in the dams so as to provide meaningful results. The prevalence of infection of *A. marginale*, *B. bigemina*, *T. mutans* and *T. parva* was estimated by determining the percentage of the 548 dams that were seropositive for each haemoparasite at the time of recruitment of the calves. The results (Table 1) suggest that the prevalences are sufficiently high to allow an estimate of colostral uptake in calves. Table 2 shows the number of different infections per dam, as judged by seropositivity. Importantly, only 64 (11.67%) of the 548 dams were seronegative for all four infections, suggesting that an indication of colostral uptake would be available for most of the calves in the study. Most dams were seropositive for either one (31.6%) or two (30.5%) parasites, with only 6.9% showing evidence of exposure to all four of the parasites. These seroprevalence figures may be an underestimate, as discussed in 3.5. The results also indicate that cattle in the region are exposed to an intensive challenge from tick-borne pathogens, in particular *B. bigemina* and *T. parva*.

### 3.2 Colostral uptake

The antibody levels in the sera of dams and calves at recruitment (3–7 days after birth) were used to estimate the number of calves that had taken colostrum. Any calf whose antibody level was greater than the cut-off value for any of the four infectious agents was considered to have received colostrum. A calf that was seronegative for any parasite and whose dam was seropositive was considered not to have received colostrum. Of the 548 dam/calf pairs in the study, 57 (10.4%) gave results that were inconsistent, where the results for one parasite indicated that the calf had received colostrum and those for another suggested the calf had not. Of the remaining 491 calves, 457 (93.08%) were judged to have taken colostrum and 17 (3.46%) appear not to have received colostral antibodies even though the dam was seropositive. In a further 17 (3.46%) cases, neither the dams nor the calves were seropositive for any of the infections and no conclusion could be drawn. The results suggest that almost all of the calves in this farming system receive colostral antibodies from the dam. It was noted that, when the farmers were questioned at the time of calf recruitment as to whether the calf had suckled the dam, 99% of the 545 farmers who answered the question responded positively.

The inconsistent results are difficult to explain. Some of these reflect borderline results, where the PP value was close to the cut-off value. In other cases, it is possible that the periparturient decrease in serum IgG_1_ levels in the dam contributed to the anomalous results (Section 3.5).

### 3.3 Duration of colostral antibody

Fig. 1 illustrates the decline in maternal antibodies in the calves for each of the four infections. The graphs show data for only those calves that had a declining PP value during the first 21 weeks to exclude calves that seroconverted during this period. It is clear that many calves were still seropositive by 16 weeks, although almost all of the calves were seronegative by 21 weeks of age. The mean half-life of colostral antibodies as indicated in the graphs varied from 5.54 weeks (*A. marginale*) to 5.56 weeks (*B. bigemina*), 6.64 weeks (*T. mutans*) and 6.42 weeks (*T. parva*). These results are important for at least two reasons. First, the interpretation of seroprevalence data generated by single time-point estimates of antibody levels will be confounded by the presence of maternal antibody in animals under 21 weeks of age. Second, it has been shown that serological responses to infection or vaccination can be impaired by the presence of maternal antibodies (Hodgins et al., 2004; Pastoret, 2007). For tick-borne diseases, this is especially relevant for *T. parva* infection, where seroprevalence following vaccination with the live sporozoite vaccine may underestimate the efficiency of vaccine administration in young animals (Babo Martins et al., 2010).

### 3.4 Benefits of receiving colostrum on survivability and growth rate

The data were used to determine if colostral uptake affected the survivability or growth rate of calves. Using univariable analysis, no significant correlation was observed between either the seropositivity status or PP value at recruitment and death due to infectious disease or to average daily weight gain.

Eighty-eight of the 548 calves that were recruited into the IDEAL study did not survive to 51 weeks of age, a mortality rate of 16.1%. Based on post-mortem examination and laboratory assays, 34 of the 88 calves were considered to have died from *T. parva* infection, indicating that East Coast fever (the disease caused by *T. parva*) was the single largest cause of death in the cohort. Univariable analysis did not disclose any significant correlation between either the seropositivity status or PP value at recruitment and death due to *T. parva*. In addition, an analysis was undertaken of the serology data from 15 calves that died of *T. parva* infection before the age of 16 weeks, the time during which maternal antibodies would be present. Of the 15 calves, 12 (80%) were seropositive for *T. parva* antibodies at recruitment, and the mean PP value of the recruitment sera from all 15 calves was 48.8. These are very similar to the results from the total population (81.0% and 48.7, respectively, Table 1). Although the sample size is small, the results indicate that antibodies play no role in protection against mortality due to *T. parva* during the early life of the calf.

By extension, these results may indicate the uptake of antibodies to all pathogens, not just the four haemoparasites analyzed, if such antibodies are present in dam sera. It is somewhat surprising that no beneficial effect on survival or growth rate was identified. However, it should be noted that the most common cause of death due to infectious disease in the cohort was East Coast fever (Thumbi et al., submitted for publication), in which antibodies are thought to play no role in protection (Muhammed et al., 1975). In addition, the small number of calves (3.46%) that were identified as not having taken colostrum may have hindered the emergence of any obvious effect from the analysis.

### 3.5 Dam vs. calf anomaly

**Fig. 2:**
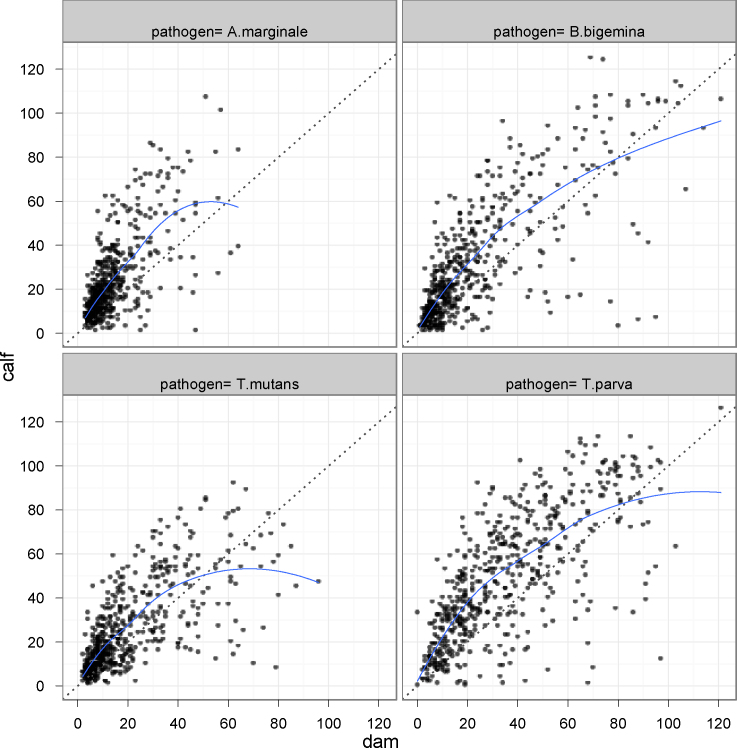
Comparison of PP results for each dam/calf pair for each haemoparasite. The dotted line is the equivalence regression line of identity (dam PP = calf PP) and the solid line is a lowess smoothed regression line summarising the mean calf/dam result.

In analyzing the data, it was observed that for each infection the number of seropositive calves was greater than the number of seropositive dams and that the calf PP value was often greater than that of the corresponding dam. This is illustrated in Table 1, which shows the percentage of seropositive animals and the mean PP value for all calf and dam sera at the recruitment sampling, for each of the four infections. An analysis of individual dam/calf pairs is shown in Fig. 2, in which the PP value for each dam is plotted against that of its calf. Most of the plotted points lie above the equivalence regression line. This phenomenon sometimes resulted in cases where the calf was seropositive and its dam was seronegative.

A possible explanation of these apparently anomalous results is based on the isotype-specific periparturient drop in the IgG_1_ levels in the dam (Sasaki et al., 1976) and the use of an anti-IgG_1_ conjugate in the ELISA used to measure the antibody levels. These factors may combine to underestimate the level of antibody in the dams at the time of recruitment in this study and suggest that care is required in interpretation of serological data in recently calved cattle.

## 4 Summary

The results presented here took advantage of the longitudinal design of the IDEAL study and the results of serology to four tick-borne haemoparasites to investigate the uptake and effects of colostrum in calves in a smallholder livestock grazing system in western Kenya. The key findings were that over 88% of the dams at recruitment had been exposed to one or more of the parasites, that almost all the calves received maternal antibodies, which were detectable in the calf until 21 weeks of age, and that the presence of the maternal antibodies had no discernible effect on the survival or growth of the calf.

## Figures and Tables

**Table 1:** The percentage of animals seropositive at recruitment for each of four haemoparasites. The mean PP is also shown (in brackets).

	*A. marginale*	*B. bigemina*	*T. mutans*	*T. parva*
Dams	29.7% (13.8)	48.9% (22.8)	34.5% (20.1)	65.2% (35.9)
Calves	59.3% (23.0)	63.0% (30.3)	48.9% (24.9)	81.0% (48.7)

**Table 2:** The number of dams seropositive for each haemoparasite, categorized according to the number of the different haemoparasite-specific antibody responses detected per dam.

	No. haemoparasite responses (dam)					
	0	1	2	3	4	Total
*A. marginale*	–	20	48	57	38	163
*B. bigemina*	–	46	102	82	38	268
*T. mutans*	–	18	58	75	38	189
*T. parva*	–	89	126	104	38	357

Total per category	64	173	167	106	38	
